# Nutritional status and quality of life among breast Cancer patients undergoing treatment in Addis Ababa, Ethiopia

**DOI:** 10.1186/s12905-023-02585-9

**Published:** 2023-08-11

**Authors:** Ruth Adam, Werissaw Haileselassie, Nabel Solomon, Yakob Desalegn, Wondemagegnhu Tigeneh, Yisihak Suga, Samson Gebremedhin

**Affiliations:** 1 School of Public Health, College of Health Sciences, Addis Ababa University, Addis Ababa, Ethiopia; 2 Department of Pathology, School of Medicine, College of Health Sciences, Addis Ababa University, Addis Ababa, Ethiopia; 3 Department of Oncology, School of Medicine, College of Health Sciences, Addis Ababa University, Addis Ababa, Ethiopia; 4 Department of Surgery, Saint Paul Hospital Millennium Medical College, Addis Ababa, Ethiopia

**Keywords:** Breast cancer, Malnutrition, Quality of life

## Abstract

**Background:**

The prevalence of malnutrition in cancer patients ranges from 30 to 60%. While it is known that malnutrition is prevalent among cancer patients, the relationship between undernutrition and quality of life among breast cancer patients has not been adequately explored. Therefore, the present study was aimed at assessing the association between undernutrition and quality of life among Breast Cancer patients under treatment in Addis Ababa, Ethiopia.

**Methods:**

A cross-sectional study on breast cancer patients under treatment at the outpatient and in-patient departments of oncology centers of two tertiary hospitals in Addis Ababa – Tikur Anbessa Specialized Hospital (TASH) and St. Paul Millennium Medical College (SPHMMC) oncology was conducted from May 12 to August 26,2020. Nutritional status was assessed using Subjective Global Assessment (SGA) screening tool. Quality of life was assessed using the European Organization for Research and Treatment of Cancer Quality of Life Questionnaire Cancer 30 (EORTC QLQ C30) a standard quality of life measurement scale for cancer patients. To determine the relationship between quality of life scores and nutritional status multivariable linear regression was fitted.

**Results:**

A total of 411 breast cancer patients with mean age of 44.4 (± 11.47) years. And 393 (95.6%) of them female were included in the study. A high prevalence of moderate 127 (30.9%) and severe 106 (25.8%) malnutrition was observed. Moderate (β = −9.21 CI (− 14.59, − 4.67)) and severe (β = −17.81 CI (− 16.6, 2 − 2.91)) malnutrition were negatively associated with the overall quality of life. Malnutrition also showed negative associations with all domains of functional status (P < 0.05) and strong positive associations with symptom scores covered in the EORTC QLQ C-30 (P < 0.05).

**Conclusion:**

This study indicated that malnutrition is a major problem among breast cancer patients and the nutritional status breast cancer patients was related to their quality of life….

**Supplementary Information:**

The online version contains supplementary material available at 10.1186/s12905-023-02585-9.

## Introduction

Breast cancer has become the leading cause of global cancer incidence, with an estimated 2.3 million new cases, representing 11.7% of all cancer cases and the fifth leading cause of cancer deaths (6.7%) worldwide in 2020 [1]. In Africa, breast cancer causes an estimated 168,690 cases and 74,072 deaths [2]. In Ethiopia, breast cancer is the most common cancer type accounting for 34% of all female cancer cases [3, 4]. Breast cancer incidence has increased from 18 to 400 new cases between 1997 and 2012 in Addis Ababa [5] Breast cancer affects young or middle age women when they also have responsibilities of raising their children and taking care of the family as a whole, it also has significant societal implications [6].

In breast cancer patients, undetected or unaddressed malnutrition leads to severe adverse outcomes and an unfavorable prognosis [7]. Malnourished breast cancer patients tolerate fewer cycles of chemotherapy and radiotherapy and are susceptible to treatment toxicities, treatment failures, and rapid progression of the disease [8]. Malnourished breast cancer patients also face repeated hospitalizations and higher treatment costs. Malnutrition also increases mortality among cancer patients by 2 to 5 times than non-malnourished cancer patients [7–9].

To improvsurvival rates and reduce potential recurrence, breast cancer patients undergo different types of treatment modalities. In Ethiopia, 83% of breast cancer patients receive chemotherapy treatment as a frontline therapy or adjuvant or palliative care. Adjuvant and neo-adjuvant chemotherapy is provided for a duration of 3 to 8 months [6]. The side effects of these treatments indirectly impact food intake or absorption of nutrients. In order to reduce the occurrence of nausea and vomiting that commences after chemotherapy sessions, prolonged fasting periods after are common in breast cancer patients. But, fasting extending beyond 48 h causes muscle-mass loss resulting in further deterioration of their nutritional status [10, 11]. Furthermore, these days, because of the critical rise of obesity, breast cancer patients are facing sarcopenic obesity a condition where loss of lean mass is veiled by adiposity\fluid retention making it harder for conventional screening methods like BMI to identify nutritional deficit [12, 13]. This renders breast cancer patients to suffer from both loss of muscle mass and increased body fat at the same time making them have many negative implications like treatment toxicity. functional incapacity, and reduced muscular strength and mobility [12, 13].

To ensure appropriate nutrition care is provided, an in-depth assessment of a patient’s nutrition status is needed, and the SGA has been developed for this purpose. The SGA was first created by Detsky et al. [14]. It’s an easy-to-use and noninvasive clinical tool that combines data from subjective and objective aspects of medical history. In our setting, the SGA tool has been validated before [15].

Quality of Life (QOL) is defined as the patient’s perspectives on their ability to live useful, meaningful, and fulfilling lives despite being burdened with disease [16]. In oncology patients, QoL is becoming a critical issue because more patients can now be treated, albeit not necessarily cured. Therefore, the expected survival of these patients is longer now than it was a few years ago, and so there is a consequent need to satisfy patients’ needs and expectations regarding their everyday life. In this respect, it is important to note that cancer patients often prefer to trade off months of survival if this is associated with a better QoL [17].

Due to advances in oncological care, the influence of nutritional status on Quality of Life has become an issue of interest and almost all newly diagnosed cancer patients believe that nutrition has a role to play in their anticancer treatment [10]. It is an instinct for every human being to value food intake in order to maintain social structure, self-esteem, and enjoyment [11].

The existing body of literature indicates that malnutrition is associated with increased morbidity and mortality globally [8, 9, 17–24]. However, its relationship with the quality of life of breast cancer patients particularly in low-income countries like Ethiopia where its prevalence is rising rapidly has not been adequately explored. There are no nutritional assessments, therapy, or counseling guidelines in Tikur Anbessa or St. Paul’s oncology centers (Two of the country’s top treatment centers). In addition, there are only a few studies [19, 22, 23] evaluating the quality of life of cancer patients in Africa and specific information on the nutritional status among breast cancer patients undergoing both radiotherapy and chemotherapy is notably lacking in Ethiopia.

The purpose of this study was to determine the relationship between nutritional status and quality of life among breast cancer patients receiving treatment at major oncology centers in Addis Ababa. The findings of this study are relevant to raise awareness of cancer patients on nutritional care and enhancing oncologists’ proficiency in achieving better assistance and improving the quality of life of their patients by providing nutritional counseling and interventions tailored to the patient’s specific needs.

## Methods

### Study design, participants and setting

This facility-based cross-sectional study was conducted at Tikur Anbessa Specialized Teaching Hospital (TASH) and St. Paul’s Hospital Millennium Medical College (SPHMMC) oncology units; the two most prominent tertiary public hospitals in Ethiopia that provide cancer treatment to referred patients from all over the country. The study evaluated breast cancer patients aged 18 years or older undergoing treatment in the two hospitals between May 12 and August 26, 2020, as in- or out-patient treatment bases. Patients were excluded if they had a severe physical or mental illness that did not allow foe full participation in the study, functionally impairing substance abuse, primary tumor site other than the breast and if weight measurement could not be obtained as weight measurement was a requirement in SGA.

### Variables and measurements

#### Outcome variable

##### Quality of life

The outcome measure of this study was the quality of life QOL. It was tested using the Amharic version of the EORTC QLQC30, which was found to be accurate and valid for evaluating QOL among cancer patients[25]. The questionnaire has 30 items with three scales and 15 different domains. The three scales used were functional, symptom scale, and general health status as a separate scale: domains such as physical, role, cognitive, emotional, and social functioning under functional scale whereas dyspnea, pain, fatigue, insomnia, appetite loss, nausea/vomiting, constipation, diarrhea, and financial difficulties under symptom scale, and general health status as a separate scale. The scoring was done according to the QOL questionnaire-C30 manual [26].

#### Nutritional status

Nutritional status was assessed by the Subjective Global Assessment (SGA), through the administration of an interviewer-based questionnaire. Subjective global assessment is a reference method used to determine the nutritional status of cancer patients [14, 27]. The best aspect of this tool is that it encompasses parameters other than just weight change and BMI alone to monitor nutritional status. Therefore, not making it confounded by alterations in body composition and fluid retention commonly associated with breast cancer. SGA is the ideal tool used in the assessment of nutritional status of breast cancer patients [18, 28–30]. SGA allows the early identification of malnourished patients, especially those with altered body composition markers due to overweight and obesity [14].

The SGA is composed of two sections: medical history and physical examination. The medical history consists of five components to be assessed; (i) change in weight loss, (ii) dietary intake, (iii) the presence of gastrointestinal symptoms, (iv) functional impairment, and (v) the disease [31].

The second part of the SGA focuses on physical evidence of malnutrition (loss of subcutaneous fat, or muscle mass, edema, or ascites). After complete medical history and physical examination, each patient was classified as well nourished (SGA-A), moderately or suspected malnourished (SGA-B,) or severely malnourished (SCA-C) [31].

### Data collection

Data was collected over 3 and a half months duration from May 12 to August 26, 2020. Patients were approached in the oncology waiting rooms and wards, the aims and objectives of the study were explained and those who agreed to participate signed the informed consent form and thumbprints were used for those who couldn’t sign. physical assessment was taken in private examining rooms as well as weight measurement.

Interviewer-based questionnaires were used to assess socio-demographic status, nutritional status, quality of life, other disease, and clinical factors. Weight measurement was also taken. socioeconomic status was assessed using household fixed asset and housing condition questions. Standard and validated data collection tools were used to collect the data. Prior to data collection pre-test was conducted at both data collection centers and subjects that were included in pre-test study were excluded from the actual study.

Information on clinical variables on tumor size, tumor grade, cancer stage, presence of metastasis, time since diagnosis, treatment intent, previous treatments, and co-morbidities were obtained from medical records. Household Food insecurity was assessed the standard tool HFIAS, a tool developed by FANTA and validated for developing countries, including Ethiopia [32, 33].

Weight was measured using digital weight scales. To measure weight, the scale was placed on a flat surface and participants were measured with minimum clothes and no shoes. It was recorded to the nearest 0.1 kg. Measurement scales were carefully handled and calibrated every day by placing iron bars before the beginning of data collection and data collectors check whether the scales are at 0.00 reading before taking each measurement. Each participant was standing on the scale feet slightly apart in the middle of the platform of the scale and the data collector recorded the weight reading. Weight of the patient before six months was recorded from the patient chart and compared to the weight the patient currently has to calculate weight loss over six months.

Data quality control Recruitment and training of research assistants Research assistants with a medical background (2 BSc and 1 MSc degree holder nurses), currently working in the study units, were recruited and trained theoretically and practically for 4 days on the protocol, study procedures including the how to get informed consent, study tools and on proper data collection using pre-tested data collection tools. Training on sociodemographic/economic status, disease related, quality of life related, and SGA and weight measurements were also provided. Before the administration of the questionnaires the research staff registered, for each patient, the clinical variables, the duration of the disease, the presence of distant metastases, and tumor burden according to TNM (tumor, node, metastasis) staging [34] from the medical records.

The pre-test was conducted to identify questions that were confusing, upsetting or containing difficult vocabulary, difficult to answer questions and to identify any other problems that the patients encountered. The respondents had some difficulty understanding questions and ratings stated in the last 2 global health status questions like: “ How would you rate your overall health status in the past week?” and “how would you rate your overall QoL in the past week?” so when these items were asked additional explanations were given. There were no upsetting questions.

All data collectors from both centers participated in standardization exercise in which they took repeated measurements of ten patients. Each measurer took two weight measurements for ten patients. Measuring equipment were tested regularly during data collection. Faulty equipment was replaced. Each scale was checked daily with a standard scale. All the technical errors of measurements were within the acceptable range.

### Sampling procedure

The sample size was determined using open Epi with the assumption of single population proportion and taking 40.1% prevalence of malnutrition from a study done in the Philippines on quality of Life and nutritional status Among cancer patients on chemotherapy [18]. At a 95% confidence level and 5% margin of error and a 10% non- response rate the sample size became 411. Thus, a total of 411 participants were included in the study. In general, a non-probability sampling technique was used to select the respondents from the two hospitals. Selection of the study participants was done using the inclusion criteria and they have received and signed an informed consent. The recruitment of participants continued until the required sample size was reached.

### Operational definitions and standard definitions

#### Standard definitions

**Cancer stage-** The stage of a cancer describes the size of the cancer and how far it has spread [35].

**Food insecurity**- a state that exists when people do not have adequate physical, social, or economic access to sufficient, safe, and nutritious food to meet their dietary needs for an active and healthy lifestyle [33].

**QoL** - a multidimensional construction that measures patients’ perception of the positive and negative aspects associated with their disease and its treatment, in at least 4 aspects: physical, emotional, psychological, and treatment-related [36].

**Tumor grade**- Cancer cells are given a grade according to how different they are to normal breast cells and how quickly they are growing [35].

### Operational definitions

**Malnutrition**- lack of proper nutrition, caused by not having enough to eat, not eating enough of the right things, or being unable to use the food that one does eat [8].

#### Well-nourished

- No decrease in food/nutrient intake; Consumption of full or >¾ – < 1 share of usual meal.
- weight loss < 5% in the past six months and.
- No/very few intermittent symptoms affecting food intake and.
- Full functional capacity and.
- No/very low increase in metabolic demand and.
- No deficit in fat or muscle mass.
- OR *an individual with criteria for SGA B but with recent adequate food intake; significant recent improvement in symptoms allowing adequate food intake; significant recent improvement in function [14].

#### Moderately malnourished

A patient fulfilling at least 2–3 criteria from the symptoms listed below; data collectors were.

instructed to place most of their judgement on weight loss and poor dietary intake and.

loss of fat mass and/or muscle loss.

- Definite decrease in food/nutrient intake- leading to consumption of 1/2–3/4 share of usual meal or < 1/2 share of usual meal but increasing appetite and consumption;
- 5 − 10% weight loss in the past 6 months without stabilization or gain and.
- mild/some symptoms persisting for 2–3 times per day in the past week affecting food intake but alleviating and.
- moderate functional deficit, loss of stamina.
- mild/moderate loss of fat mass; while assessing triceps (muscle that runs down the back of the long bone of the upper arm) using fingers; fingers are closer than normal and presence of loose fitting skin around the triceps.
- mild/moderate loss of muscle mass; some protrusion of the clavicle (collar bone) but not all the way along. Slight depression on temple area. Slight protrusion of the acrominon process on shoulder area [37].
- OR *an individual meeting criterion for SGA C but with improvement (but not adequate) of oral intake, recent stabilization of weight, decrease in symptoms affecting oral intake, and stabilization of functional status [14].

#### Severely malnourished

A patient fulfilling at least 2–3 criteria from the symptoms listed below; data collectors were.

instructed to place most of their judgement on weight loss and poor dietary intake and.

loss of fat mass and/or muscle loss. If the patient had considerable edema, ascites, or tumor mass, data collectors were told to be less influenced by the amount of weight loss.

- Severe deficit in food/nutrient intake; consumption of < 1/2 share of usual meal, no change or decreasing or Starvation (< 1/4 of usual meal).
- weight loss > 10% which is ongoing;
- Some or all symptoms affecting food/ nutrient intake experienced 3 times a day or above in the past week or.
- Severe loss of functional ability or bedridden.
- Mild to moderate increase in metabolic demand (moderate stress).
- OR *recent significant signs of fat and/or muscle loss; very little space between fingers or fingers touch while assessing triceps, hollowed or depressed temple, protruding/prominent clavicle, prominent /square looking shoulder bones [14, 37].

The raters were instructed to be less sensitive and more specific in their assignment of rankings. That is, if the features which might influence the rater to assign a B rank (as opposed to an A rank) are equivocal or doubtful, an A rank is appropriate. Similarly, a C rank implied definite findings of severe malnutrition.

### Data management and analysis

Data was entered and coded using epi data version 4.6.0 and exported to the STATA software (release 16.0, Stata Corporation, College Station, TX, USA) for data analysis. Data was cleaned for inconsistencies and missing values. All variables with missing data were reported. Variables were assessed for normality, linearity, homoscedasticity, multicollinearity, and for outliers. Descriptive statistical analysis was conducted using frequency, percentage and mean (SD) to describe the study population by explanatory variables. A relative socioeconomic status was derived for the study households using a principal component analysis (PCA). Households were then categorized into quintiles ranging from the poorest to the richest. PCA was calculated as commonly done in national Demographic and Health Surveys. Dummy variables were created for nutritional status as it has three categories. We also fitted fifteen separate multiple linear regression models and factors potentially associated with QoL: age, sex, marital status, education level, occupation, family size, lifestyle factors, wealth status, monthly income, financial support, food security, cancer stage, tumor grade, metastasis, time since diagnosis, treatment type, comorbidity were adjusted to determine the association between nutritional status and each scale of quality of life. All variables having a p-value of ≤ 0.2 in the bivariate analysis were further entered into multi-variable regression. Multi-variable linear regression was performed. The result of the β-coefcient was used for the interpretation of the strength of prediction of the independent variables to the domains of QoL. In all cases, statistical significance was set at a p-value of < 0.05.

### Ethical considerations

The study received ethical approval from the Research Ethical Committee of the School of Public Health, Addis Ababa University (SPH/599/2020). During the data collection, written informed consent was obtained from each patient after explaining the objectives of the study. To maintain anonymity identifiers like names were not included in the questionnaire. All measures to maintain human rights including informed consent, the right to participate in the study, the right to privacy and confidentiality and right to prevention from any type of harm were taken into consideration.

## Results

### Demographics

A total of 411 patients participated in the study. The mean (± SD) age of the study participants was 44.34 ± 11.47 years (range 20–68 years). A great number of the respondents were females 393 (95.6%) and around a quarter 103 (25.06%) of the participants had no formal education, while only 43 (10.4%) had attended tertiary education. More than half of the patients were married 261 (63.6%) and 242 (58.8%) came from Addis Ababa. See Table 1 for full demographic and clinical details of participants.

**Table 1:** Socio-demographic characteristics of breast cancer patients diagnosed at TASH and SPMMC 2020

Variables (n = 411)	Category	Frequency (n)	Percent (%)
**Gender**	Male	18	4.3
	Female	393	95.6
**Religion**	Orthodox	246	59.5
	Protestant	96	23.3
	Muslim	54	13.1
	Catholic	10	2.4
	Other	5	1.2
**Educational Status**	No formal Education	103	25.0
	Primary Level (1–8)	90	21.9
	Secondary Level (9–12)	104	25.3
	Technical/Vocational	71	17.2
	Higher (university or above)	43	10.4
**Marital Status**	Married	261	63.6
	Single	69	14.6
	Widowed	45	10.9
	Divorced	35	8.5
	Separated	9	2.2
**Occupation**	Housewife	165	40.0
	Civil servant	96	23.4
	Non-government employee	48	11.7
	Merchant	42	10.2
	Farmer	28	6.8
	Daily laborer	19	4.3
	Other	13	3.1
**Residence**	Addis Ababa	242	58.8
	Out of Addis Ababa	169	41.1
**Living Arrangement**	Living with a partner	258	62.7
	Living with children	66	16.0
	Living with a parent/s or siblings	59	14.3
	Living alone	28	6.8
**Family size**	< 5	218	53.0
	≥ 5	193	46.9
Wealth quantiles	Poorest	83	20.1
	Poor	82	19.9
	Middle	82	19.9
	Rich	82	19.9
	Richest	82	19.9
**Food security**	Food secure	174	42.3
	Mildly food insecure	63	15.3
	Moderately food insecure	123	29.9
	Severely food insecure	51	12.4

***Religion: Other: - Jehovah’s Witnesses, Adventists, ethnic religion***

### Clinical characteristics of participants

Among the study subjects, 357(86.8%) were diagnosed for the first time while54 (13.1%) had recurrence (breast cancer that came back after the initial treatment). More than half 236 (57.4%) of the subjects were diagnosed within the past year, while 5 years have passed since the diagnosis of 19 (4.6%) of patients. Three- quarters of the patients 131 (31.8%) had stage 4 cancers, while only 30 (7.3%) were diagnosed with stage 1. Only 67 (16.3%) of the subjects had chronic co-morbidities and the most common was hypertension 25 (37.3%). See Table 2 for full clinical details of participants.

**Table 2:** Clinical characteristics of breast cancer patients treated at TASH and SPMMC, 2020

Variables	Category	Frequency (n)	Percent (%)
**Time since diagnosis (months)**	< 12	236	57.4
	12–24	52	12.6
	25–36	58	14.1
	37–48	31	7.5
	49–60	15	3.6
	> 60	19	4.6
**Cancer stage (n = 372)**	Stage I	30	7.3
	Stage II	87	21.1
	Stage III	124	30.1
	Stage IV	131	31.8
	Not reported	39	9.4
**Tumor size**	Tx (Not assessed)	10	2.4
	T1 (< 2 cm)	60	14.5
	T2 (2-5 cm)	150	36.4
	T3 (> 5 cm)	78	18.9
	T4 (Any size with extension to chest wall)	113	27.4
**Tumor Grade (n = 287)**	Grade I	53	12.9
	Grade II	83	20.1
	Grade III	116	28.2
	Grade IV	35	8.5
	Not reported	124	30.1
**Comorbid disease (n = 67)**	Hypertension	25	37.3
	Diabetes mellitus	14	20.9
	HIV AIDS	15	22.3
	Asthma	6	8.9
	Hypertension and Diabetes mellitus	7	10.4
**Treatment modalities** **(previous)**	Surgery only	53	12.9
	Chemotherapy only	59	14.3
	Chemotherapy and radiotherapy	17	4.1
	Chemotherapy and surgery	69	50.8
	Chemotherapy, radiotherapy and surgery	142	16.7
**Treatment patient is** **currently on**	Surgery	53	12.9
	Chemotherapy	249	60.3
	Radiotherapy	21	4.8
	Hormonal therapy	51	12.4
	Follow up (anti-pain, zoledronic acid)	37	6.5
**Treatment Intent**	Palliative	134	33.8
	Curative	276	66.1
**Metastasis at initial diagnosis**	Present	149	36.2
	Absent	262	63.7
**History of recurrence**	Yes	54	13.1
	No	357	86.8

### Financial status of partcipants

Regarding the financial status of the study participants, Monthly mean household income was 4604.16 (± 186.41). While 298 (72.5%) of them had their own source of income, 147 (35.7%) of the participants had additional financial support from family members or relatives. Out of the 147 (35.7%) participants that had additional financial support, 93 (22.6%) of them got this support regularly and only 33 (8.03%) thought that this support was enough to meet their needs. Table 3 presents the financial status of the study participants.

**Table 3:** Financial status of breast cancer patients treated at TASH and SPMMC,2020

Variables	Category	Frequency (n-411)	Percent (%)
Monthly income, mean(SD)		4604.16 (± 186.41)	
Presence of own income	Yes	298	72.5
	No	113	27.5
Presence of financial support	Yes	147	35.7
	No	264	64.3
Who provides financial support	Family members	124	30.17
	Relatives	11	2.68
	Neighbors	4	0.97
	Friends	6	1.46
	Other people	2	0.49
	No financial support	264	64.3
Is the financial support regular?	Yes	93	22.6
	No	54	13.2
	No financial support	264	64.3
Is the financial support enough?	Not enough	73	17.8
	Fair	41	9.9
	Enough	33	8.0
	No financial support	264	64.3

### Nutritional status of participants

**Fig. 1:**
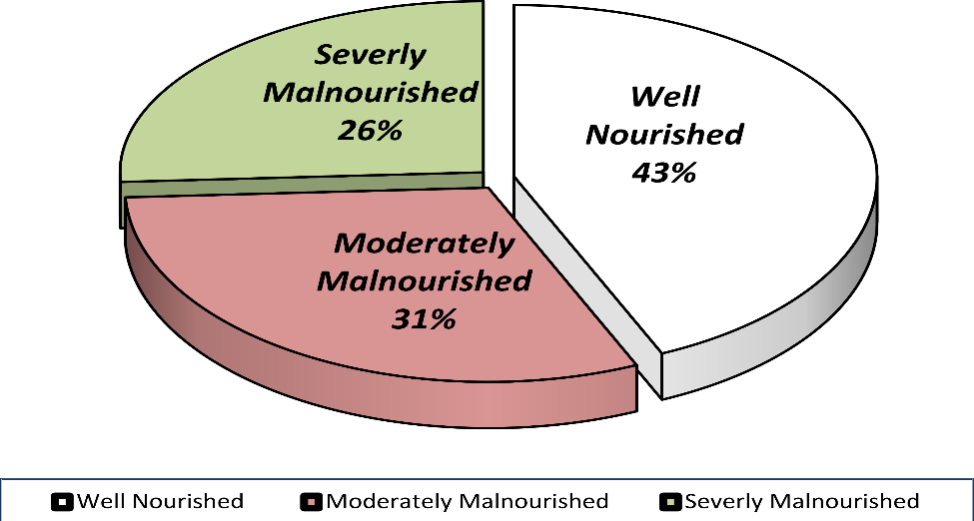
Magnitude of malnutrition in breast cancer patients treated at TASH and SPMMC, 2020

According to their subjective global assessment scores, the patients were divided into 3 groups. Nearly half 178 (43.3%) of the study participants were well nourished (SGA A) while 127 (30.9%) were moderately malnourished (SGA B) and 106 (25.79%) were severely malnourished (SGA C). Weight loss was also prevalent in the study subjects with 21 (5.17%) having lost more than 15% of their body weight in the past six months followed by 28 (6.9%) who’d lost between 10 and 15% and 52 (12.27%) subjects losing between 5 and 10% of their previous body weight in the past six months. (See Fig. 1).

### Profile of quality of life scale scores

The QoL of the study participant was measured by the domains in the functional and symptom scales of the EORTC-QLQ C-30 and by one summary measure (GHS/QoL). Among the functional scale domains; the first domain with the highest mean score was cognitive functioning with a mean and standard deviation of 76.85 (SD = 26.87) followed by emotional functioning 70.42 (SD = 28.95)). The lowest mean among functional scales was observed in social functioning with mean of 55.03 (SD = 36.26). The domain with the highest mean among the symptom scales was fatigue with mean and standard deviation of (M = 48.98 (SD = 29.03)) while the lowest score was observed in nausea and vomiting (M = 21.65 (SD = 31.60)). The mean global health status of the study participants was 61.33 with a standard deviation of 20.83. Table 4 presents the QOL profiles of the study participants.

**Table 4:** The level of quality of life of breast cancer patients treated at TASH and SPMMC,2020

Dimensions	Mean (n = 411)	SD	CI for mean	
			Lower	Upper
Global health status/ overall quality of life				
GHS/QoL	61.3	20.8	59.3	63.3
Functional scales				
Physical functioning	61.7	26.7	59.1	64.3
Role functioning	58.0	35.7	54.5	61.4
Emotional functioning	70.4	28.9	67.6	73.2
Cognitive functioning	76.8	26.8	74.2	79.4
Social functioning	55.0	36.2	51.5	58.5
Symptom scales				
Fatigue	48.9	29.0	46.1	51.8
Nausea & vomiting	21.6	31.6	18.5	24.7
Pain	46.7	31.5	43.6	49.7
Dyspnea	21.7	32.8	18.5	24.9
Insomnia	24.3	34.9	20.9	27.7
Appetite loss	42.9	37.8	39.2	46.5
Constipation	25.6	35.8	22.1	29.1
Diarrhea	7.7	22.2	5.5	9.8
Financial difficulties	57.7	40.8	53.7	61.7

### The relationship between nutritional status and quality of life

There was a significant relationship between nutritional status and quality of life. It was found that in terms of malnutrition mean QoL level of those who were moderately malnourished and severely malnourished was lower than those who were well nourished β − 9.21 (CI − 13.59, − 4.67) and β − 17.81 (CI − 22.83, − 12.95) respectively, taking well-nourished as a referent group. Similarly, severe malnutrition also showed a strong negative relationship with PF (β= −20.01 p < 0.001) taking well-nourished as a referent group. The most noteworthy associations were also observed between nutritional status and fatigue, nausea and vomiting, and appetite loss symptom scores. Results of the regressions showed that fatigue scores increased by 20.03 and 27.31 in moderately and severely malnourished patients (β = 20.03, p < 0.001) and (β = 27.31, p < 0.001) respectively as opposed to well-nourished ones. And patients in the moderately & severely malnourished groups had pain scores 18.54 & 31.73 higher than well-nourished groups (β = 18.54, p < 0.001) and (β = 31.73, p < 0.001) respectively. Appetite loss also showed a negative association with moderate (β = 27.80, p < 0.001) and severe (β = 41.32, p < 0.001) malnutrition taking well-nourished as a referent group (Table 5).

In Table 5, we present a separate multivariate linear regression model fitted to determine the relationship between the scales of quality of life and nutritional status. This accounts for age, sex, marital status, education level, occupation, family size, lifestyle factors, wealth status, monthly income, financial support, food security, cancer stage, tumor grade, metastasis, time since diagnosis, treatment type and comorbidities.

**Table 5:** Bivariable and multivariate linear regressions models of nutritional status as a predictive factor of quality of life domain scores in breast cancer patients (n = 411) treated at TASH and SPMMC, 2020

	Moderately malnourished (vs. well-nourished)		Severely malnourished (vs. well nourished)	
	Bivariable analyses	Multivariable analyses	Bivariable analyses	Multivariable analyses
	β (95% CI)	β (95% CI) ^a^	β (95% CI)	β (95% CI) ^a^
Overall QOL	−14.2 (− 18.29, − 10.11)**	−9.21 (− 13.59, − 4.67)**	−26.16 (− 30.48, − 21.84)**	−17.81 (− 22.83, − 12.95)**
Physical functioning	−13.43 (− 18.95, − 7.9)**	−11.08 (− 16.87, − 5.28)**	−28.37 (− 34.21, − 22.54)**	−17.89 (− 26.53, − 13.49)**
Role functioning	−19.45 (− 26.84, − 12.05)**	−14.10 (− 21.75, − 6.13)**	−37.77 (− 45.58, − 29.96)**	−28.02 (− 37.01, − 19.03)**
Emotional functioning	−19.05 (− 25.18, − 12.93)**	−14.62 (− 21.99, − 8.84)**	−25.06 (− 31.53, − 18.59)**	−15.99 (− 25.52, − 10.54)**
Cognitive functioning	−15.04 (− 20.79, − 9.3)**	−12.01 (− 18.09, − 5.93)**	−22.73 (− 28.8, − 16.67)**	−17.94 (− 24.61, − 11.27)**
Social functioning	−22.83 (− 30.61, − 15.05)**	−13.72 (− 19.94, − 4.29)**	−27.93 (− 36.15, − 19.71)**	−24.49 (− 24.14, − 6.68)**
Fatigue	21.78 (15.89, 27.68)**	20.03 (13.61, 26.45)**	31.2 (24.97, 37.42)**	27.31 (20.22, 34.39)**
Nausea and Vomiting	16.63 (10.64, 22.62)**	14.80 (8.2, 21.42)**	44.06 (37.74, 50.38)**	40.51 (33.23, 47.79)**
Pain	22.23 (15.78, 28.68)**	18.54 (11.45, 25.63)**	33.6 (26.78, 40.41)**	31.73 (23.84, 39.61)**
Dyspnoea	15.57 (8.43, 22.7)**	15.63 (7.68, 23.59)**	24.23 (16.69, 31.77)**	19.59 (10.62, 28.46)**
Insomnia	13.36 (5.6, 21.12) **	14.63 (6.07, 23.17)**	20.23 (12.04, 28.43)**	20.05 (10.68, 29.42)**
Appetite loss	30.28 (22.95, 37.61)**	27.80 (19.87, 35.72)**	48.02 (40.28, 55.76)**	41.32 (32.64, 50.00)**
Constipation	11.9 (3.9, 19.9)**	13.26 (5.14, 21.38)**	18.3 (9.85, 26.75)**	17.03 (8.17, 25.88)**
Diarrhea	7.08 (2.21, 11.95)**	6.42 (1.30, 11.55)**	16.29 (11.15, 21.44)**	13.43 (7.69, 19.17)**
Financial difficulties	24.08 (15.28, 32.87)**	12.22 (4.56, 21.89)**	30.94 (21.65, 40.23)**	11.65 (1.94, 21.36)**

*β, regression coefficient; CI, confidential interval; QoL, quality of life*

*P < 0.05, **P < 0.01

^a^ Adjusted for age (years), sex, marital status, education level, occupation, family size, lifestyle factors, wealth status, monthly income, financial support, food security, cancer stage, tumor grade, metastasis, time since diagnosis, treatment type, comorbidity

## Discussion

This facility-based study was conducted on two of the largest hospitals with oncology units in the country. The Findings of this study showed us that more than half of the patients were moderately or severely malnourished, and malnutrition decreased the functional scores of quality of life of these patients while increasing/worsening of symptoms.

According to our study, moderate or severe malnutrition was prevalent in 233 (57%) of the patients which is much lower than studies conducted on breast cancer patients in Indonesia [38] Libya [23] and Algeria [22]. But, higher than a study done in Brazil [39] that had only 60 (24%) of its participants moderately and severely malnourished.

Our results are also different from a study done in France [24] which revealed that only 18.3% of the breast cancer patients were malnourished but the study only used BMI and weight loss as nutritional measurements which are not indicated as sole nutrition assessment tools for cancer patients [23, 40–43] as they are prone to be highly affected by water retention affecting weight or presence of excess body fat masking the loss of lean body mass sometimes leading to malnourished cancer patients having a normal BMI despite having 10–20% weight loss in 6 months [23]. But our findings are similar to a study conducted in 13 countries in Latin America [20], and studies from South Korea (94) and Mexico [44].

Weight loss in the past 6 months was also prevalent among our study population with greater than 10% weight loss being recorded in 49 (12%) of the participants which happens to be in line with studies from New Mexico [44] and France [24]. But, our results are much lower than a study done in Algeria [22] which found that 49 (29.3%) of its population had weight loss greater than 10%, but this could be due to the difference in time of seeking care between the populations, compared to the former population in which 144 (86.6%) of the study subjects had locally advanced or metastasized tumors at diagnosis, our population only had 149 (36.2%) and it’s well established that patients with more advanced cancers are more malnourished [22].

As nutritional status is a determining factor for quality of life [29], the present study highlighted the importance of nutritional assessment among breast cancer patients in light of its associations with functional status, medical symptoms experienced and overall quality of life. Malnutrition, either in its moderate or severe form measured by SGA showed a direct and significant association with worse functional & symptom scores as well as QoL. The negative association of malnutrition and unintentional weight loss > 10%, with functional scales and QoL was demonstrated in many previous literatures [28, 40, 43, 45].

Overall quality of life/ global health status showed a significant relationship with nutritional status. Moderately and severely malnourished patients had 9.21, 17.81 (p < 0.001) lower QoL scores compared to not malnourished patients respectively. Studies from the U.S., Mexico, Austria, France and Malaysia [40, 43, 46, 47] all confirm that impaired quality of life has association with malnutrition. However, in a study from Malesia [48], it was found that dietary intake was not associated with QoL.

Physical functioning was negatively associated with malnutrition according to our results. This was consistent with results from a study in Indonesia [19] and Brazil [49] in which malnutrition was related to decreased physical function and increased loss of energy. A systematic review by Hidding et al. [50] also suggested that initial treatments involving mastectomy and axillary lymph node dissection can contribute to muscle dysfunction and limitations in doing daily activities pointing out the long-term decrease in physical functioning.

A particular aspect of our study worth considering is the association between role functioning and malnutrition similar to a study from Belgium [51]. Malnourished women with breast cancer are also susceptible to more infections, decreased physical mobility & fatigue. These symptoms added to the burden imposed by the cancer diagnosis on their jobs and domestic tasks force them to require prolonged sick leaves, early retirements etc., making them unable to continue their employment and diminish their ability to do domestic work around the house and care for their loved ones. These in turn limit the role they play in their family and place of work. For most, this change from being needed to needing someone is very difficult to accept and leads to decreased feelings of autonomy [52].

Another noteworthy association was also observed between cognitive functioning and malnutrition. Malnourished patients had much lower cognitive scores compared to well-nourished subjects. This finding is supported by studies from France and Malesia [40, 41]. It is all too well known that improvised nutritional status decreases physiologic functions, leading to cognitive impairment [53]. However, a prospective study that evaluated the association of nutritional parameters with QoL found associations between nutritional status and all other functional scales except cognitive functioning [54].

Emotional functioning, as one study indicated, meant the capability to enjoy life [52]. In the current study malnourished patients had significantly reduced emotional functioning scores concurrent with the findings from a study done in Brazil [49] and China [55]. Treatment induced side effects dispose breast cancer patients to decreased appetite, energy intake and eventually cause malnutrition, which has negative consequences on their physical and emotional function [56]. These complications deprive the patients of their ability and willingness to continue treatments. Patients admitted to occasions in which they had lost the will to live, with most under-nourished patients reporting feeling down, irritated at the smallest things and depressed. These feelings are an indicator of emotional instability [52, 55].

Social functioning was negatively associated with malnutrition, published data have highlighted that the incidence of GI symptoms such as anorexia, nausea and vomiting, stomatitis, and mucositis are related to cancer as well as treatments major causes of malnutrition in these patient groups but this relationship could also be bi-directional, meaning malnutrition could also in turn cause these symptoms [57]. It is also likely that the manifestations of the above mentioned symptoms could be a reason for the lack of motivation to perform daily activities making them unable to successfully care for themselves, Malnutrition could also restrict these individuals in different social activities by inducing fatigue and mood disorders [21]. These findings are similar to the significant negative relationship observed between malnutrition & social functioning in the present study.

Concerning symptom scores, fatigue was found to be another distressing symptom next to appetite loss that showed a significant linear relationship with malnutrition (P < 0.001) which is also true for a study done in Brazil that explained it as when nutritional status is compromised muscle mass loss leads to body weakening. And when this is topped by the naturally expected physiological and functional decline caused by aging, it reduces the capability of movement while increasing fatigue dramatically [21]. Appetite loss, nausea and vomiting, fatigue and diarrhea could be causes or effects of malnutrition in breast cancer patients and could lead to consequent deterioration in overall quality of life and functional scales, treatment side effects such as toxicity leading to a dosage reduction, delay or suspension of treatment, and, finally, in worse prognosis [43, 57].

A cross-sectional study conducted on gynecological cancer patients from which 68.7% were with breast cancer diagnosis, found that out of all the symptom scales, appetite loss and fatigue were strongly associated with malnutrition. Especially in those patients currently on chemotherapy regimen as the drugs used caused significant taste alterations which put the patients at risk of losing weight and developing many kinds of nutritional deficits [47]. An Algerian study also classified appetite loss as the most common symptom in breast cancer patients affecting their food intake and was present in 15–25% of the patients with advanced stage [22].

Similar results in all symptom scores except diarrhea and dyspnoea were demonstrated in a previous study in patients with cancer [48]. However, different findings were also seen in a Malesian study [58] where dyspnea, diarrhea and nausea & vomiting were the least complained symptoms with values almost close to zero [41]. But, it should be remarked that quality of life domains rely very much on personal values or perceptions, whereby each domain could be regarded differently across individuals as well as populations and different types of treatment regimens and drugs are used across different countries resulting in different symptom severities.

A bidirectional relationship between malnutrition and symptom scores is possible as the symptoms such as nausea and vomiting, appetite loss and diarrhea that predict a decreased quality of life are also the symptoms that predispose breast cancer patients to be malnourished [22].

As a significant strength, this study attempted to address a major public health problem which is the interaction between malnutrition and quality of life. The study incorporated all dimensions of quality of life scales which helped to assess its association with nutritional status on a wider scope. The study also used locally validated and well-known standard tools which made it easier to compare its findings with other international studies. It also included a homogeneous population (one cancer type) which made it possible for the findings to represent that cancer type.

However, the results of this study should be interpreted with caution due to the following limitations. First and foremost, the cross-section design nature of the study makes it not possible identify which is the cause and which is the consequence: nutritional status and quality of life. Secondly, previous weight measurements found on the medical records and sometimes asked from the patients’ when there are no records are not properly done using calibrated measurement scales, clothes weren’t removed, the possible presence of edema was not recorded, etc. making these weight measurements below standard and known only approximately. But, to correct this problem weight loss or gain greater than 2 kg was considered significant. Third, the nutritional status, as well as QoL measures, were based on the patients’ self-report rather than direct observation of dietary intakes, presence of symptoms, and living conditions making most assessments subjective. This might introduce some bias. Although there are many advantages to employing observations and laboratory tests instead of questionnaires, these techniques are not cost-effective, especially in large samples like the present study. which is why these studies relied on patient reports and physical examinations.

## Conclusion and recommendations

### Conclusion

This study revealed that the prevalence of malnutrition in the breast cancer population is very large and under-nourished patients have worsened functional, emotional, social, role and physical scores of QoL compared to well-nourished patients. Our study adds to the body of evidence that confirms the relationship between nutritional status and QoL in breast cancer patients and it is hoped that outcomes of this study may pave the way for betterment of health policies aimed at improving nutritional status and quality of life of breast cancer patients.

### Electronic supplementary material

Below is the link to the electronic supplementary material.

Additional File 1: Pre-test sample of patient response sheet

Additional File 2: Technical error of measurement for weight

## Data Availability

The datasets generated and analyzed during the current study are available from the corresponding author on a reasonable request.

## Abbreviations

BC: Breast Cancer
BMI: Body Mass Index
CA: Cancer
CF: Cognitive functioning
DLMIC: Developing and Middle-Income Countries
EF: Emotional functioning
EORTC: European Organization for Research and Treatment of Cancer
EORTC QLQ-C30: European Organization for Research and Treatment of Cancer Quality of Life Questionnaire Cancer 30
GI: Gastrointestinal
GLOBOCAN: Global organization board of cancer association network
HRQOL: Health related quality of life
KG: Kilograms
PCA: Principal component analysis
PF: Physical functioning
QOL: Quality of Life
RF: Role functioning
SF: Social functioning
SGA: Subjective Global Assessment
SPHMMC: Saint Paul’s Hospital Millennium Medical College
TASH: Tikur Anbessa Specialized Hospital
TNM: Tumor, Nodes, Metastases
WHO: World health organization
