# Supplementary material for: Nutritional status and quality of life among breast Cancer patients undergoing treatment in Addis Ababa, Ethiopia
Source: BMC Womens Health. 2023 Aug 11;23:428. doi: 10.1186/s12905-023-02585-9 (PMC10422709; doi:10.1186/s12905-023-02585-9)
Supplement: Supplementary file 1 — Additional File 1: Pre-test sample of patient response sheet [file 12905_2023_2585_MOESM1_ESM.docx]

**Pre-test sample of patient response sheet**

Questionnaire number--------------

yes, No Comment

A, difficulty

B, confusing

C, difficult words

D, upsetting words

E, how would you ask

this question

**Pre-test respondents socio-demographic and clinical characteristics**

**Socio-Demographic characteristics of pre-test respondents in TASH and SPMMC, Addis Ababa, 2020**

| **Variable** | **Category** |  | **Frequency (n=20)** | **Per cent (%)** |
| --- | --- | --- | --- | --- |
| **Sex** | Male |  | 9 | 45 |
|  | Female |  | 11 | 55 |
| **Educational status** | No formal education |  | 5 | 25 |
|  | Able to read and write |  | 3 | 15 |
|  | Primary education |  | 6 | 30 |
|  | Secondary education |  | 3 | 15 |
|  | College and above |  | 3 | 15 |
| **Occupation** | Farmer |  | 3 | 15 |
|  | Civil servant |  | 5 | 25 |
|  | House wives |  | 6 | 30 |
|  | Non-government employee |  | 1 | 5 |
|  | Student |  | 2 | 10 |
|  | Merchant |  | 3 | 15 |
| **Region** | Addis Ababa |  | 12 | 60 |
|  | Out of Addis Ababa |  | 8 | 40 |
|  |  |  |  |  |

**Clinical characteristics of pre-test respondents in TASH and SPMMC, Addis Ababa, 2020**

| **Variables** | **Category** | **Frequency(n=20)** | **Percent** | |
| --- | --- | --- | --- | --- |
|  |  |  | **(%)** |  |
| **Tumor grade** | Grade 1 | 5 | 25 |  |
|  | Grade 2 | 5 | 25 |  |
|  | Grade 3 | 4 | 20 |  |
|  | Grade 4 | 6 | 30 |  |
|  | Chemotherapy | 4 | 20 |  |
|  | Surgery | 6 | 30 |  |
| **Type of treatment** | Chemotherapy and surgery | 2 | 10 |  |
|  |  |  |  |  |
|  | Chemotherapy and Radiotherapy | 2 | 10 |  |
|  |  |  |  |  |
|  | Chemotherapy, Surgery, and Radiotherapy | 6 | 30 |  |
| **Stage** | Stage I | 4 | 20 |  |
|  | Stage II | 2 | 10 |  |
|  |  |  |  |  |
|  | Stage III | 2 | 10 |  |
|  | Stage IV | 12 | 60 |  |
| **Treatment intent** | Curative | 5 | 25 |  |
|  | Palliative | 15 | 75 |  |
|  |  |  |  |  |

| **Marital status** | Married | 11 | 55 |
| --- | --- | --- | --- |
|  | Single | 3 | 15 |
|  | Divorced | 5 | 25 |
|  | Widowed | 1 | 5 |
| **Age, mean(SD)** |  | 45.50(10.75) |  |
