# Supplementary material for: Nutritional status and quality of life among breast Cancer patients undergoing treatment in Addis Ababa, Ethiopia
Source: BMC Womens Health. 2023 Aug 11;23:428. doi: 10.1186/s12905-023-02585-9 (PMC10422709; doi:10.1186/s12905-023-02585-9)
Supplement: Supplementary file 2 — Additional File 2: Technical error of measurement for weight [file 12905_2023_2585_MOESM2_ESM.docx]

Technical error of measurement for weight

TASH

|  |  | Measurer: 1 | |  |  |  | Measurer: 2 | |  |  |
| --- | --- | --- | --- | --- | --- | --- | --- | --- | --- | --- |
| subject | 1st | 2nd | Difference(d) | | d^2^ | 1st | 2nd | Difference(d) | d^2^ |  |
| number | measurement | measurement |  |  |  | measurement | measurement |  |  |  |
|  |  |  |  |  |  |  |  |  |  |  |
| 1 | 76.5 | 76.2 |  | 0.3 | 0.09 | 75.9 | 75.6 | 0.3 | 0.09 |  |
| 2 | 84.5 | 84.2 |  | 0.2 | 0.04 | 84.7 | 84.4 | 0.3 | 0.09 |  |
| 3 | 66.4 | 66.8 |  | -0.4 | 0.16 | 66.1 | 65.8 | 0.3 | 0.09 |  |
| 4 | 60.3 | 60.0 |  | 0.3 | 0.09 | 60.6 | 60.2 | 0.4 | 0.16 |  |
| 5 | 94.3 | 94.0 |  | 0.3 | 0.09 | 94.0 | 94.3 | -0.3 | 0.09 |  |
| 6 | 53.7 | 53.4 |  | 0.3 | 0.09 | 53.3 | 53.2 | 0.1 | 0.01 |  |
| 7 | 62.5 | 62.3 |  | 0.2 | 0.04 | 63.1 | 63.2 | -0.1 | 0.01 |  |
| 8 | 59.2 | 59.0 |  | 0.2 | 0.04 | 59.3 | 59.1 | 0.2 | 0.04 |  |
| 9 | 45.3 | 45.6 |  | -0.3 | 0.09 | 45.0 | 45.3 | -0.3 | 0.09 |  |
| 10 | 63.7 | 63.4 |  | 0.3 | 0.09 | 63.9 | 63.6 | 0.3 | 0.09 |  |
| Absolute Intra Observer TEM √∑ di^2^/ 2N = 0.2 Kg | | | | | | Absolute Intra Observer TEM = √∑ d^2^/ 2N= 0.19 kg | | | |  |

Inter observer TEM = 0.2156

Standard deviation = 1.48

Coefficient of reliability = 97.5 %

Where:

∑d2 = summation of deviations raised to the second power

N = number of volunteers measured

i= the number of deviations

SPMMC

|  |  | Measurer: 1 | |  |  |  | Measurer: 2 | |  |  |
| --- | --- | --- | --- | --- | --- | --- | --- | --- | --- | --- |
| subject | 1st | 2nd | Difference(d) | | d^2^ | 1st | 2nd | Difference(d) | d^2^ |  |
| number | measurement | measurement |  |  |  | measurement | measurement |  |  |  |
|  |  |  |  |  |  |  |  |  |  |  |
| 1 | 66.3 | 66.5 |  | -0.2 | 0.04 | 65.1 | 65.2 | -0.1 | 0.01 |  |
| 2 | 57.8 | 57.7 |  | 0.1 | 0.01 | 57.6 | 57.4 | 0.2 | 0.04 |  |
| 3 | 63.5 | 63.7 |  | -0.2 | 0.04 | 63.2 | 62.3 | -0.1 | 0.01 |  |
| 4 | 80.6 | 80.7 |  | 0.1 | 0.01 | 80.5 | 80.7 | -0.2 | 0.04 |  |
| 5 | 55.8 | 56.0 |  | 0.2 | 0.04 | 55.2 | 55.4 | -0.2 | 0.04 |  |
| 6 | 48.6 | 48.8 |  | -0.2 | 0.04 | 48.8 | 48.9 | -0.1 | 0.01 |  |
| 7 | 62.5 | 62.1 |  | 0.4 | 0.16 | 63.1 | 63.0 | 0.1 | 0.01 |  |
| 8 | 59.6 | 59.3 |  | 0.3 | 0.09 | 59.9 | 59.0 | -0.1 | 0.01 |  |
| 9 | 62.2 | 62.3 |  | -0.1 | 0.01 | 62.6 | 62.5 | 0.1 | 0.01 |  |
| 10 | 58.7 | 58.8 |  | -0.1 | 0.01 | 58.2 | 58.2 | 0 | 0 |  |
| Absolute Intra Observer TEM √∑ di^2^/ 2N = 0.16 kg | | | | | | Absolute Intra Observer TEM = √∑ d^2^/ 2N= 0.096kg | | | |  |

Inter observer TEM = 0.2241

Standard deviation = 1.35

Coefficient of reliability = 98.42 %

Where:

∑d2 = summation of deviations raised to the second power

N = number of volunteers measured

i= the number of deviation
